# Consumers' acceptance of the first novel insect food approved in the European Union: Predictors of yellow mealworm chips consumption

**DOI:** 10.1002/fsn3.2716

**Published:** 2022-01-18

**Authors:** Ruxandra Malina Petrescu‐Mag, Hamid Rastegari Kopaei, Dacinia Crina Petrescu

**Affiliations:** ^1^ Faculty of Environmental Science and Engineering Babes‐Bolyai University Cluj‐Napoca Romania; ^2^ Department of Rural Development Management Faculty of Agriculture Yasouj University Yasouj Iran; ^3^ Faculty of Business Babes‐Bolyai University Cluj‐Napoca Romania; ^4^ Department of Marketing, Innovation and Organization Faculty of Economics and Business Administration Ghent University Ghent Belgium

**Keywords:** disgust, food consumption drivers, insect‐based foods, perceptions, yellow mealworm

## Abstract

Climate and environmental‐related challenges are high on the agenda of the European Union (EU). One priority is to redesign the existing food system into a more sustainable one, where the link between healthy people and a balanced environment is considered. The EU bets on the role of insect farming in supporting the transition toward healthier and future‐proof diets. Following this orientation, we investigated consumers' attitude toward yellow mealworm chips (YMC) and identified the predictors of YMC consumption. The causal relationships between constructs were explored using the structural equation modeling (SEM) based on partial least squares (PLS) using SmartPLS software. The perceived lower environmental impact of YMC compared to meat was the most appreciated characteristic of YMC. The study identified five predictors of YMC consumption, among which the perceived characteristics of YMC have the strongest influence on the consumption probability. Against the expectations of the authors, disgust with the accidental encounter of insects in foods did not influence the probability of eating YMC. Age was another predictor of YMC consumption. It is known that food preferences and eating behaviors are mainly developed during childhood and tend to manifest in adult life. Consequently, it can be inferred that acceptance and preference for insect‐based foods (IBF) should be stimulated from early childhood. Finally, practical implications are advanced as possible solutions to overcome the obstacles toward YMC consumption.

## Graphical abstract

The EU bets on the insect farming role in supporting the transition towards healthier and future‐proof diets. Following this orientation, we investigated consumers’ attitude towards yellow mealworm chips (YMC). The study identified five predictors of YMC consumption, among which the perceived characteristics of YMC have the strongest influence on consumption probability.

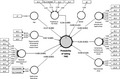

## 1 INTRODUCTION

The nexus between food, environment, and climate change has been largely explored in the scientific literature (Kemper & Ballantine, 2019; Laestadius et al., 2016; Petrescu et al., 2017; Stoll‐Kleemann & Schmidt, 2017). Therefore, the economic benefits brought about by livestock and poultry production that contributes to approximately half of the global agricultural economy (Qian et al., 2018) contrast with the emissions of high nitrogen, phosphorus, and other pollutants in the sector (Godfray et al., 2018). Overall, the agricultural sector generates almost 23% of global greenhouse gas emissions (Kamilaris et al., 2018), and animal husbandry is one of the contributors to today's environmental problems (Steinfeld et al., 2006). Therefore, meat production and consumption, attitudes toward reducing meat consumption, and the search for sustainable alternative protein sources are under scrutiny when researching the impact of agriculture on the environment.

Within this reality, a better performance regarding emissions, land and water use, or the high content of quality protein, vitamins, minerals, and fats (Baiano, 2020; Gravel & Doyen, 2020; da Silva Lucas et al., 2020; Van Huis, 2013), or cost‐effective opportunities (Van Huis, 2013) promote insect‐based food (IBF) as a sustainable supplement to existing protein sources (Cho & Ryu, 2021; Fischer & Steenbekkers, 2018). The Farm‐to‐Fork Strategy (European Commission, 2020), which is at the heart of the European Green Deal (European Commission, 2019), shows that climate and environmental‐related challenges are high on the agenda of the European Union (EU), aiming at redesigning the existing food system into a more sustainable one, where the link between healthy people and a balanced environment is considered. The Farm‐to‐Fork Strategy bets on the role of insect farming in supporting the transition toward healthier and future‐proof diets. A new emerging market for insects or insect‐based ingredients is expected to develop in the coming years in the EU (Sogari et al., 2019). In this way, in May 2021, “dried yellow mealworms” (species *Tenebrio molitor*) were approved as the first novel insect food under EU Regulation 2015/2283. Roncolini et al. (2019) reported that mealworm larvae are sweet with a nutty flavor and cocoa smell. There is a strong interest in the use of the dried yellow mealworms as a food source both for humans and animals (Rumbos et al., 2020), since they have high protein and lipid content, they are a good source of amino acids (Finke, 2015), and they were found to be safe for human consumption if allergenic potential is taken into account by people who are at risk of allergies (EFSA Panel on Nutrition et al., 2021).

Although around 2000 species of insects are included in the daily diet of about 3000 ethnic groups worldwide (Deroy et al., 2015; Tao & Li, 2018), most European consumers are unfamiliar with IBF and react with disgust (Lombardi et al., 2019). A step toward reducing human impacts on the environment is through changing people's food choices (Graham & Abrahamse, 2017). This is all the more important because, according to McLeod (2011), meat demand will increase by 73% between 2010 and 2050 (compared to the 2010 level when the global meat demand was 286.1 million tons, according to the Food and Agriculture Organization of the United Nations (FAO) estimations (n.d.)). Following Thøgersen's (2021) review, where he refers to Moran et al.s' (2020) study, it is inferred that the EU has the potential to reduce its carbon footprint by approximately 25%, and changes in the consumption pattern (28% of the total) are the most impactful. That is why it is not negligible to direct efforts toward changes in attitudes and behavior.

**FIGURE A1:**
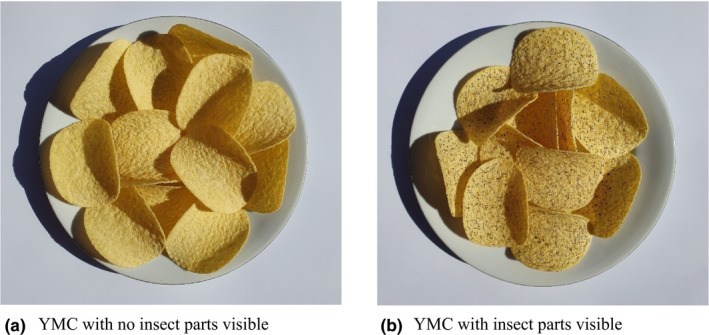
Yellow mealworm chips (YMC) photographs shown to respondents in the questionnaire. (a) YMC with no insect parts visible. (b) YMC with insect parts visible

The present study aims to advance knowledge on the acceptance by Romanian consumers of an IBF, namely yellow mealworm chips (YMC). The investigated consumers were informed that the chips presented in the pictures (Figure A1) contained yellow mealworms processed into powder. Chips were chosen as the investigated food category because they are one of the most popular foodstuffs (Mesías & Morales, 2015) that can be found in a wide variety (e.g., shape, form, flavor, greasiness). Even if reliable statistics on the food sector of chips (import, exports, consumption/capita, domestic production) are not available for Romania, it was found that in the first 3 months of 2020, the chips market increased by 25% in value and 14.2% in volume compared to the same period in 2019; and, in general, the local market of salty snacks (which includes chips) records a growth rate twice the European average (around 5%–6%) (according to the Progresiv portal (2020)). However, this increase is contextual since 2020 was a pandemic year, and snack consumption was considered a necessary “pampering”, with consumers spending most of their time at home. Furthermore, research developed in different European countries showed that neophobia and disgust are two barriers to accepting insect food (Hartmann et al., 2015; Orkusz et al., 2020). Consumers' neophobia and sensory barriers can be overcome by incorporating insects into familiar products such as bread, biscuits, and pastries and/or associating the insects with preferred flavors (Megido et al., 2016; Mishyna et al., 2020; Wilkinson et al., 2018). Therefore, another reason for choosing yellow mealworm chips (YMC) was linked to reducing prejudices about IBF, which could be done by not presenting insects in a real biological form (Orkusz et al., 2020), but as an “invisible” ingredient in a familiar product that may increase their acceptance (Khalil et al., 2021). Therefore, an insect snack is considered a more acceptable way for consumers to start consuming insects than an insect‐based meal.

Despite the increasing literature dedicated to IBF, some aspects remain less investigated. “How do Romanian consumers perceive YMC?”, “What are the drivers of Romanian consumers toward the YMC consumption?”, “Do the propensity to new foods, the relationship between the attitude toward food healthiness and food choice, disgust toward the accidental encounter of insects in food, perceived characteristics of YMC, drivers of YMC consumption, gender, and age influence the probability of eating YMC by Romanians?”. These are the three research questions (RQs) of the study, to which the following main objectives respond. First, to reveal the attitude of Romanian consumers toward YMC. The attitude was investigated considering the consumers' assessment of the characteristics of YMC, the consumption drivers, and the consumption probability. Second, to identify the predictors of YMC consumption. To the authors' best knowledge, this is the first study that investigates Romanian consumers' attitudes and their consumption probability of a specific insect‐based product: yellow mealworm chips.

## 2 LITERATURE REVIEW

The unfamiliarity with IBF is one of the barriers to consumption and market development in Western societies (Roma et al., 2020). This may depend on the sociocultural background, disgust reactions, health risk perceptions associated with its consumption, or neophobia (Hartmann et al., 2015; Pliner & Hobden, 1992; Roma et al., 2020; Tan et al., 2015; Verbeke, 2015). Practically, food neophobia, meaning the fear of consuming unfamiliar foods, significantly contributes to a decreased dietary variety and quality (Jaeger et al., 2017, 2021). When new food is introduced on the market, consumers tend to have negative feelings about it (Dolezalova, 2015). Disgust is closely related to fear of contamination (Jensen & Lieberoth, 2019). Various researches (Olatunji et al., 2007; Ruby et al., 2015) indicate that the insect disgust of Western consumers reflects this fear of contamination. Meyer et al. (2021) offered an overview of the potential chemical food safety hazards (e.g., heavy metals, dioxins, mycotoxins) that might be present in IBF. It was confirmed that factors related to insect species, life stage, and source of the contaminant affect the accumulation of contaminants in insects. Existing research on mealworms and additives derived from them shows that they must carry an allergy warning (Grau et al., 2017). The yellow mealworm can induce sensitization through inhalation or skin contact with transmembrane (TM) proteins followed by the clinical manifestation of allergic symptoms in previous nonallergic persons (Bernstein et al., 1983; Broekman et al., 2016; Downs et al., 2016; Garino et al., 2020; Senti et al., 2000). Broekman et al. (2017) and Garino et al. (2020) warned that crustaceans‐allergic patients are at risk of mealworm and other insect allergies. A comprehensive overview of the insect food allergy, including mealworms, is provided by de Gier and Verhoeckx (2018). They reported various cases of allergy after insect ingestion, proteins causing insect allergy, including cross‐reactive proteins, and the possibility of heat processing and in vitro digestion to reduce the allergic reaction (which is not eliminated, however) (Van Broekhoven et al., 2016). In this context, IBF should be included in the list annexed to Regulation (EU) 1169/2011 (the FIC Regulation) (2011) on the labeling of food and food products to protect the health of allergic consumers (Garino et al., 2020). Therefore, further research on the safety and allergenicity of IFB is needed to gain knowledge about the real risks associated with IBF consumption.

Besides the perceived fear of the risks associated with the IBF consumption, Rozin and Fallon (1980) named other main causes for food rejection: dislike of its sensory characteristics and disgust, mostly connected to the nature and origin of the product. One of the strongest emotional reactions associated with IBF consumption is the disgust, the yuck factor (Nemeroff & Rozin, 1989; Verbeke, 2015). Disgust is triggered by various visual, tactile, and olfactory attributes of food, either experienced or imagined, and motivates consumption behavior against IBF (Curtis et al., 2011; Jensen & Lieberoth, 2019). For example, the most common reason for not tasting IBF was the disgust factor (Sogari et al., 2017), which was reported by students from Italy studying Gastronomy and Food Science. Another study of 248 university students from Italy investigated disgust and other potential attributes that could influence the consumption of IBF. It revealed that a large part of the sample (31.9%) was very disgusted by the idea of eating IBF (Lorini et al., 2021). In Germany, the willingness to consume the insect burger and buffalo worms was also strongly influenced by disgust, with 41.9% of the participants willing to consume an insect burger, and only 15.9% of them willing to consume the main ingredient of the insect burger—buffalo worms (Lammers et al., 2019).

Insect visibility in food is a direct trigger of disgust and negatively influences the willingness to eat IBF (Hartmann et al., 2015). Naranjo‐Guevara et al. (2021) reported that visual appearance was a critical point for accepting IBF by Dutch and German students, and the “invisibility” of insects was one of the predictors of their willingness to incorporate insects into their diets. As regards the association of insects with known flavors, the invisible inclusion of insects in foods (insect flour in cookies, insect protein in energetic bars) is below a greater anticipated acceptance of IBF (Gmuer et al., 2016; Megido et al., 2014, 2016). The degree of processing of the insect ingredient partly influenced the ratings of Swiss consumers, with “deep‐fried crickets” being assessed more negatively than cricket “flour” or “bits” products (Gmuer et al., 2016). There is also a downside of these insect‐processed products. Hartmann and Siegrist (2017) reported that insect‐processed foods such as cookies or chips contain only a small amount of insects that will not reduce the amount of meat consumed. Consequently, the impact on health and the environment will not be significant.

In general, concern for the environment is decisive in developing sustainable consumption behavior (Mascarello et al., 2015; Petrescu et al., 2020). Although many consumers express their environmental concerns, they do not act consistently as such (Vermeir et al., 2020). There is often a gap between favorable environmental attitudes and the actual purchase of sustainable food exists (Aschemann‐Witzel & Zielke, 2017). Consumers of IBF are perceived to be more environmentally friendly than meat consumers (Hartmann et al., 2018). Therefore, IBF marketing strategies most often endorse messages related to environmental sustainability and health aspects, as happens in Germany (Lammers et al., 2019; Müller et al., 2016). On the contrary, other research considers that advertising strategies that emphasize positive but distant goals such as health or environmental benefits are ineffective (Berger et al., 2018). Furthermore, in a study on German consumers, Lammers et al. (2019) found no significant correlation between sustainability consciousness and the willingness to eat IBF. The existing studies show that health concerns stimulate the interest in alternative foods (Jones & Beynon, 2021), which might be applicable to IBF, as well (in which the nutritional value is one of the main benefits that could compensate citizens' reluctance toward its consumption). However, most consumers in developed countries are reluctant to consume IBF in any context other than as a novelty item (Hartmann & Siegrist, 2017; Verbeke, 2015).

Curiosity has also been reported to be a motivating factor for IBF consumption (Sogari, 2015; Yen, 2009). Consumers eating IBF out of curiosity seek gourmet or adrenalin experiences and are more likely to introduce IBF as a future source of protein (Dolezalova, 2015). Furthermore, it seems that consumers with high sensation food‐seeking tend to have a lower food neophobia (Alley & Potter, 2011). Furthermore, consumer knowledge of insect food and previous experience with eating insects are associated with an increased likelihood of consuming IBF. In a study by Piha et al. (2018) focused on consumers from Northern and Central Europe (Finland, Sweden, Germany, and Czech Republic), consumer knowledge influences their willingness to buy insect food.

Consumers' acceptance ratings of IFB are not always an adequate predictor of product market success (Gmuer et al., 2016; King & Meiselman, 2010). Therefore, price can significantly influence customer satisfaction, as buyers always pay attention to pricing when assessing the value of the product and service (Vasić et al., 2019; Zeithaml, 1988). However, the price and availability of IBF have not been in the foreground of consumer acceptance research (House, 2016). Next, conformity, seen as other people's (friends, family) food evaluations, can influence the likelihood of accepting IBF. Social influence has been reported in various studies as a relevant factor toward approaching or not IBF (Menozzi et al., 2015; Sogari et al., 2017).

## 3 METHODOLOGY

### 3.1 Sample selection and description of the variables

Data were collected through face‐to‐face interviews. A county was randomly selected in each development region of Romania, plus the capital city. In each county, a city was randomly selected as follows. The list with cities in a county (*i*) was created and the cities were numbered from 1 to *n_i_* (n being the total number of cities in county *i*); a random number from the range (1, …, *n_i_*) was generated in Excel, and the city with the corresponding number was included in the study. Two shopping points (supermarket or pharmacy) were chosen randomly in each of the eight cities. The number of persons interviewed in each county was proportional to the county population. An interview was requested for every fifth person who passed through the shopping center entrance. The final sample included 394 persons, 79% women and 21% men. The questionnaire was pretested on a sample of 50 people and adjusted. The final version contained an Introduction and nine sections. The Introduction explained the purpose of the questionnaire, what the YMC were, informed consent for participation, and the general data protection regulations (GDRP). The questionnaire was anonymous, participation was voluntary, and participants did not receive any financial reward for their responses.

The YMC were defined for participants as chips that contained thermally dried yellow mealworms in the form of powder. They were also explained that Art. 10 of Regulation (EU) 2015/2283 allowed to place on the market dried yellow mealworm (*T. molitor* larva) as a novel food.

The questionnaire was then divided into seven sections (Table A1). Following Verbeke's (2015) model, food neophobia and consumer attitudes toward the health characteristics of food in general, reflected in their food choices, were included in the questionnaire (Verbeke, 2015). Therefore, the first section contained four statements related to food neophobia (selected from the Pliner and Hobden scale (1992)). The opposing attitude to food neophobia is the propensity to new foods. Because two out of three were positively formulated about trying new foods, this variable was named “Propensity to new foods” in the present study. The second section included two statements about the relationship between consumers' attitude toward food healthiness and their food choice (Roininen et al., 1999). The third section included three questions about the disgust toward the accidental encounter of insects in food because disgust toward insects in food was reported as an important factor that influenced the IBF consumption (Wendin & Nyberg, 2021). The three tested accidental encounters were selected prior to the implementation of the survey as follows. A question was asked to 100 people (different from the final sample used in this study), namely, to share the most frequent situations in which they found insects in their food (cooked and fresh, excluding ingredients such as flour), in descending order (starting with the most frequent food/meal where they found insects). The average frequency was calculated for each situation, and the three most frequent situations were included in the final questionnaire. Because various food characteristics represent motivations for food consumption, the perceived characteristics of the investigated product (YMC, in the present case) were included in the questionnaire. Thus, the fourth section contained a list of nine characteristics of insect food. Drivers toward YMC consumption were also included to observe which of them are indicated by consumers as YMC consumption drivers in their case. The characteristics and consumption drivers of YMC were obtained through two focus group discussions with 10 consumers each. Finally, the fifth section tested 16 drivers of YMC consumption. The sixth section tested the probability of eating YMC, and a picture with insect chips (Figure A1a) was shown to the interviewed persons. The image in Figure A1a showed real chips, while Figure A1b was digitally edited by authors to resemble chips with small insect parts because these were not available on the market. The seventh section asked about demographics (such as age and gender).

### 3.2 Model description

The basic hypothesis in this study is that the perceived probability of eating YMC can be directly affected by age, drivers related to convenience, nutritional, environmental, emotional, sensorial, health, and peers' example, disgust toward accidental encounter of insects in foods, the relationship between the attitude of consumers about food healthiness and their food choice, perceived characteristics of YMC, and propensity to new foods. The causal relationships between constructs were explored by multiple group analysis (MGA) and structural equation modeling (SEM) based on at least partial references using SmartPLS (3.2.8). Hair et al. (2016) suggested the use of PLS‐SEM when the research model has constructs with features such as a single indicator formative and reflective. The critical model parameters and criteria in PLS‐SEM analysis and their recommended thresholds followed Hair et al. (2016) because their work is a reference point in statistics, being one of the most used and cited. Furthermore, exploratory factor analysis (EFA) was used to identify which variables are perceived to be similar and, therefore, to identify factors (such as “convenience, nutritional, and environmental drivers”, “emotional, sensorial, and health drivers”, and “peers example driver”). EFA results indicated values within the recommended limits (Kaiser–Meyer–Olkin (KMO) = 0.941, Approx. chi‐square = 7877.3, and Sig. = 0.0001) and, thus, the method was successful in identifying the factors.

## 4 RESULTS

### 4.1 General food attitudes and YMC attitudes

The results show that the YMC are evaluated around the midpoint (Table 1) and consumers' propensity to new foods is slightly above average. Food healthiness is important in the food choices of respondents, but only slightly above the neutral point. The findings indicate that people are disgusted when they accidentally find insects in their food. However, disgust did not receive a high score. Most YMC features obtained scores around the midpoint, with two exceptions. In one case, people consider that YMC have a much lower environmental impact than meat. In the other case, they believe that visible yellow mealworm parts in chips are very repulsive. The most important consumption driver is appearance, followed by odor and taste. YMC consumption has a low probability, close to the undecided point.

**TABLE 1:** Consumers' evaluation of yellow mealworm chips (YMC)

Variables	Items	Average scores; % of total sample
Propensity to new foods (opposed to food neophobia)	I always look for and try new foods	4.78
	If I don't know what food contains, I don't eat it (reverse coded in analyses)	3.14^a^
	At parties, meetings with friends, I try new food	4.60
	I eat almost anything	4.00
Relationship between consumers' attitude toward food healthiness and their food choice	How I choose a food does not depend on how healthy that food is	4.83^a^
	I eat whatever I like, and I don't care how healthy or not food is	4.60^a^
Disgust toward the accidental encounter of insects in food	Worms in soup	3.93
	Worms in fresh cherries	4.11
	Worms in polenta	3.35
Perceived characteristics of YMC	Taste	3.44
	Odor	3.55
	Texture	3.36
	Aspect when yellow mealworm parts are visible (Figure A1b)	2.30
	Aspect when yellow mealworm parts are not visible (Figure A1a)	4.16
	Characteristic of being appetizing	2.90
	Nutrient intake offered	4.01
	Consumption effect on human health	4.15
	Environmental impact compared to meat	5.40
Drivers of YMC consumption: ‐ Emotional, sensorial, and health drivers	Taste	4.74
	Odor	4.80
	Appearance	4.96
	Healthiness	4.48
	Therapeutic effect	4.58
	Curiosity	4.06
‐ Convenience, nutritional, and environmental drivers	Affordable price	3.20
	Easiness to consume	3.39
	Availability in stores	3.67
	Nutrient intake offered	4.09
	Lower water consumption than meat	3.58
	Lower environmental pollution than meat production	4.20
	Reduction of number of slaughtered animals	4.28
‐ Peers' example drivers	My family consumes it	3.10
	My friends consume it	2.70
	Many other people consume it	2.63
Probability of eating YMC	Probability of eating YMC (Figure A1a)	3.98
Age	Age	30.57
Gender	Men	21%
	Women	79%

^a^ Calculated with reversed codes. The original scores were 4.86, 3.17, and 3.40, respectively.

### 4.2 Measurement model

Due to the difference in the evaluation criteria recommended for reflective and formative constructs, these two types of constructs were evaluated separately. For reflective constructs, outer loadings, composite reliability (CR), average variance extracted (AVE), and heterotrait–monotrait (HTMT) criteria were used, and they are indicated in Tables 2 and 3. As can be seen, all outer loadings of reflective constructs are >0.6, which shows that all outer loadings are suitable. The outer loadings reflect the convergence between the indicators of each construct. Moreover, all CR and AVE values have significant levels, which means that all indicators of each construct have an acceptable relationship together (Table 2). Furthermore, the heterotrait–monotrait (HTMT) criteria shown in Table 2 have values below the threshold of 0.8 suggested by Hair et al. (2016). This means that all indicators belong to their own construct and have a deep divergence from other constructs (Hair et al., 2016).

**TABLE 2:** Measurement properties of reflective constructs

Constructs	Indicators (label)	Outer loading
Disgust with the accidental encounter of insects in foods (CR = 0.917, AVE = 0.787)	Worms in soup (q3_1)	0.924
	Worms in fresh cherries (q3_2)	0.909
	Moths in polenta/bread/semolina (q3_3)	0.825
Relationship between consumers' attitude regarding food healthiness and their food choice (CR = 0.807, AVE = 0.681)	How I choose a food does not depend on how healthy that food is (q2_1)	0.942
	I eat whatever I like, and I don't care how healthy or not food is (q2_2)	0.689
Age (CR = 1, AVE = 1)	Age (q7)	1.000
Gender (CR = 1, AVE = 1)	Gender (q8)	1.000
Probability of eating YMC (CR = 1, AVE = 1)	Probability of eating YMC (q6)	1.000

Abbreviations: AVE, average variance extracted; CR, composite variability; YMC, yellow mealworm chips.

**TABLE 3:** Heterotrait–monotrait (HTMT) distribution of reflective constructs

	Age	Disgust with the accidental encounter of insects in foods	Gender	Relationship between consumers' attitude regarding food healthiness and their food choice	Probability of eating YMC
Age	–	–	–	–	–
Disgust with the accidental encounter of insects in foods	0.231	–	–	–	–
Gender	0.153	0.135	–	–	–
Relationship between consumers' attitude regarding food healthiness and their food choice	0.165	0.151	0.084	–	–
Probability of eating YMC	0.133	0.413	0.122	0.115	–

Abbreviation: YMC, yellow mealworm chips

Regarding the formative construct, the outer weights, the outer loading, and the variance inflation factor (VIF) were used. Hair et al. (2016) suggested that the outer weight should be significant; however when an indicator's outer weight is nonsignificant but its outer loading is high (i.e., above 0.50), the indicator should be interpreted as absolutely important. In addition, the VIF must have a value below 5 to be considered acceptable; otherwise, it must be removed from the construct. In Table 4, it can be observed that all constructs have the necessary conditions to be present in the model.

**TABLE 4:** Measurement properties of formative constructs

Constructs	Indicators (label)	Outer weight	Sig.	Outer loading	VIF
Propensity to new foods	I always look for and try new foods (q1_1)	0.418	0.003	0.750	1.700
	If I don't know what food contains, I don't eat it (q1_2 recoded)	0.383	0.000	0.516	1.075
	At parties, meetings with friends, I try new foods (q1_3)	0.289	0.037	0.754	1.759
	I eat almost anything (q1_4)	0.364	0.002	0.747	1.343
Perceived characteristics of YMC	Taste (q4_1)	0.083	0.210	0.638	2.131
	Odor (q4_2)	−0.014	0.840	0.531	2.046
	Texture (q4_3)	−0.049	0.457	0.501	1.869
	Aspect when insect parts are visible (q4_4)	0.101	0.109	0.543	1.630
	Aspect when insect parts are not visible in YMC (q4_5)	0.442	0.000	0.807	1.749
	Characteristic of being appetizing (q4_6)	0.443	0.000	0.845	2.208
	Nutrient intake offered (q4_7)	−0.049	0.587	0.671	2.936
	Consumption effect on human health (q4_8)	0.185	0.071	0.687	2.621
	Environmental impact compared to meat (q4_9)	0.259	0.000	0.379	1.077
Emotional, sensorial, and health drivers	Taste (q5_1)	0.014	0.856	0.643	2.267
	Odor (q5_2)	0.214	0.045	0.671	3.844
	Appearance (q5_3)	−0.106	0.270	0.571	3.444
	Healthiness (q5_4)	0.717	0.000	0.960	2.818
	Therapeutic effect (q5_5)	0.009	0.909	0.738	2.347
	Curiosity (q5_6)	0.280	0.000	0.759	1.668
Convenience, nutritional, and environmental drivers	Affordable price (q5_7)	0.018	0.870	0.752	2.237
	Easiness to consume (q5_8)	−0.226	0.069	0.761	3.637
	Availability in stores (q5_9)	0.527	0.000	0.900	3.039
	Nutrient intake offered (q5_10)	0.302	0.004	0.892	1.233
	Lower water consumption than meat (q5_11)	0.038	0.692	0.821	1.499
	Lower environmental pollution than meat production (q5_12)	0.362	0.001	0.889	1.923
	Reduction of number of slaughtered animals (q5_13)	0.079	0.357	0.760	0.708
Peers' example driver	My family consume it (q5_14)	0.600	0.000	0.918	1.857
	My friends consume it (q5_15)	0.425	0.027	0.852	2.276
	Many other people consume it (q5_16)	0.105	0.587	0.820	2.710

Abbreviations: VIF, variance inflation factor; YMC, yellow mealworm chips.

The Standardized Root Mean Square Residual (SRMR) is a model fit measure in PLS. Henseler et al. (2014) introduced the SRMR which measures the squared discrepancy between the observed correlations and the model‐implied correlations, as a means to validate a model. This measure was implemented in the SmartPLS 3 software. Also, Garson (2016) showed that SRMR is a measure of approximate fit of the researcher's model. It measures the difference between the observed correlation matrix and the model‐implied correlation matrix. In other words, the SRMR reflects the average magnitude of such differences, with the lower SRMR being a better fit. By convention, a model has a good fit when SRMR is less than 0.08 (Hair et al., 2016; Hu & Bentler, 1998). Garson (2016) used the more lenient cutoff of less than 0.10. For the present paper, the stricter threshold of 0.08 was adopted to evaluate the model fit (Hair et al., 2016; Hu & Bentler, 1998). The SRMR value in this study was equal to 0.051, which indicates that the model highly fits the data (Hair et al., 2016). Therefore, the research model with the mentioned constructs was finally evaluated. Other studies also followed the recommendation to have a SRMR value for PLS path models less than 0.08 to achieve a good fit. They reported results of 0.07 SRMR for consumers' intention to visit restaurants that serve edible insects (Ali & Ali, 2022). Comparatively, in the present study, the model fit indicated by the SRMS was better than in the study of Ali and Ali (2022).

### 4.3 Structural model

**FIGURE 1:**
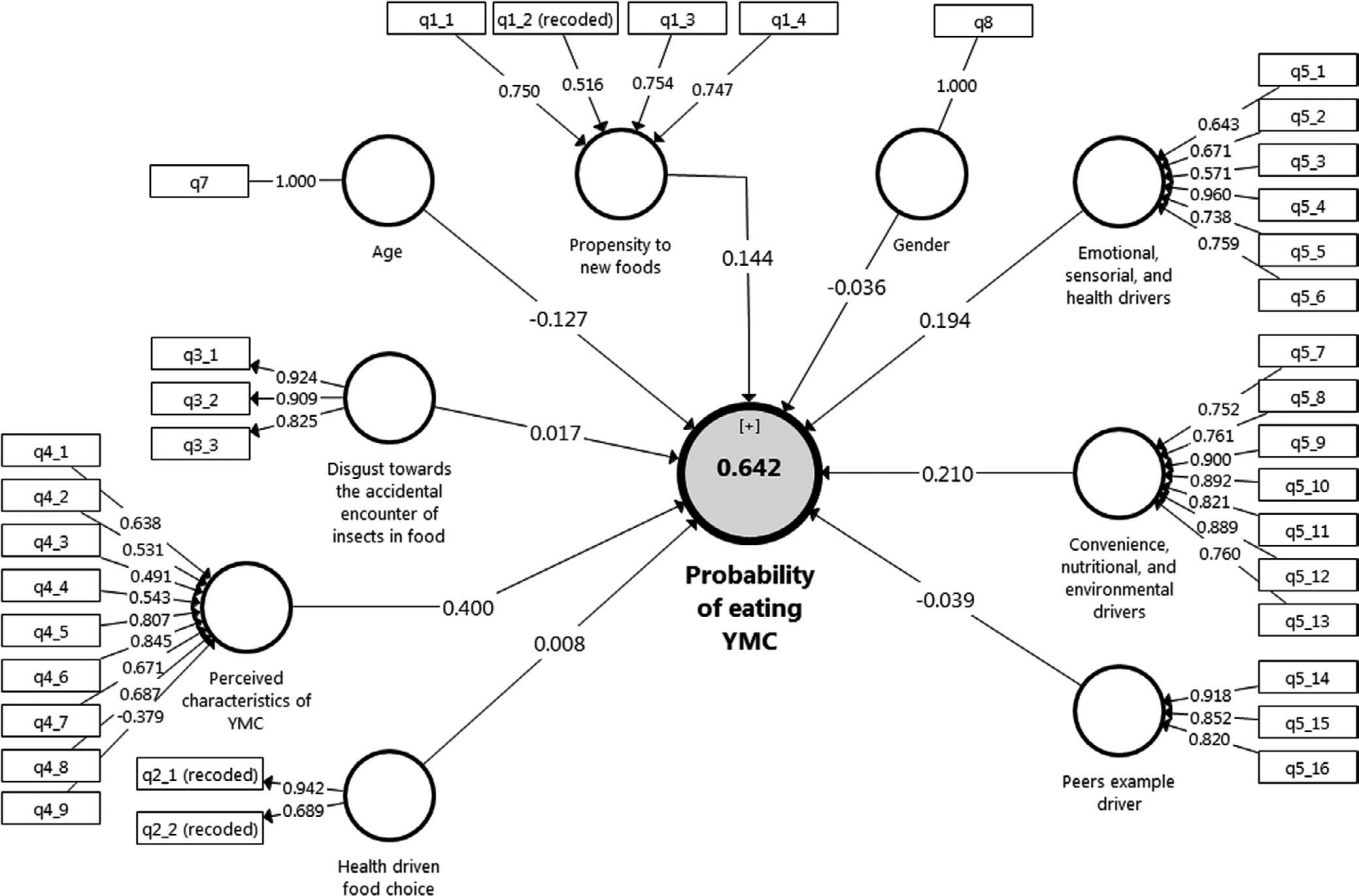
Measurement model to predict the probability of eating yellow mealworm chips (YMC)

The model parameters are presented in Figure 1. Thus, the outer loadings (the values on the arrows between rectangles and small circles), the path coefficients (the values on the arrows from small circles to big circles), and *R* ^2^_adj_. (the value in the big circle) are visible in Figure 1.

**FIGURE 2:**
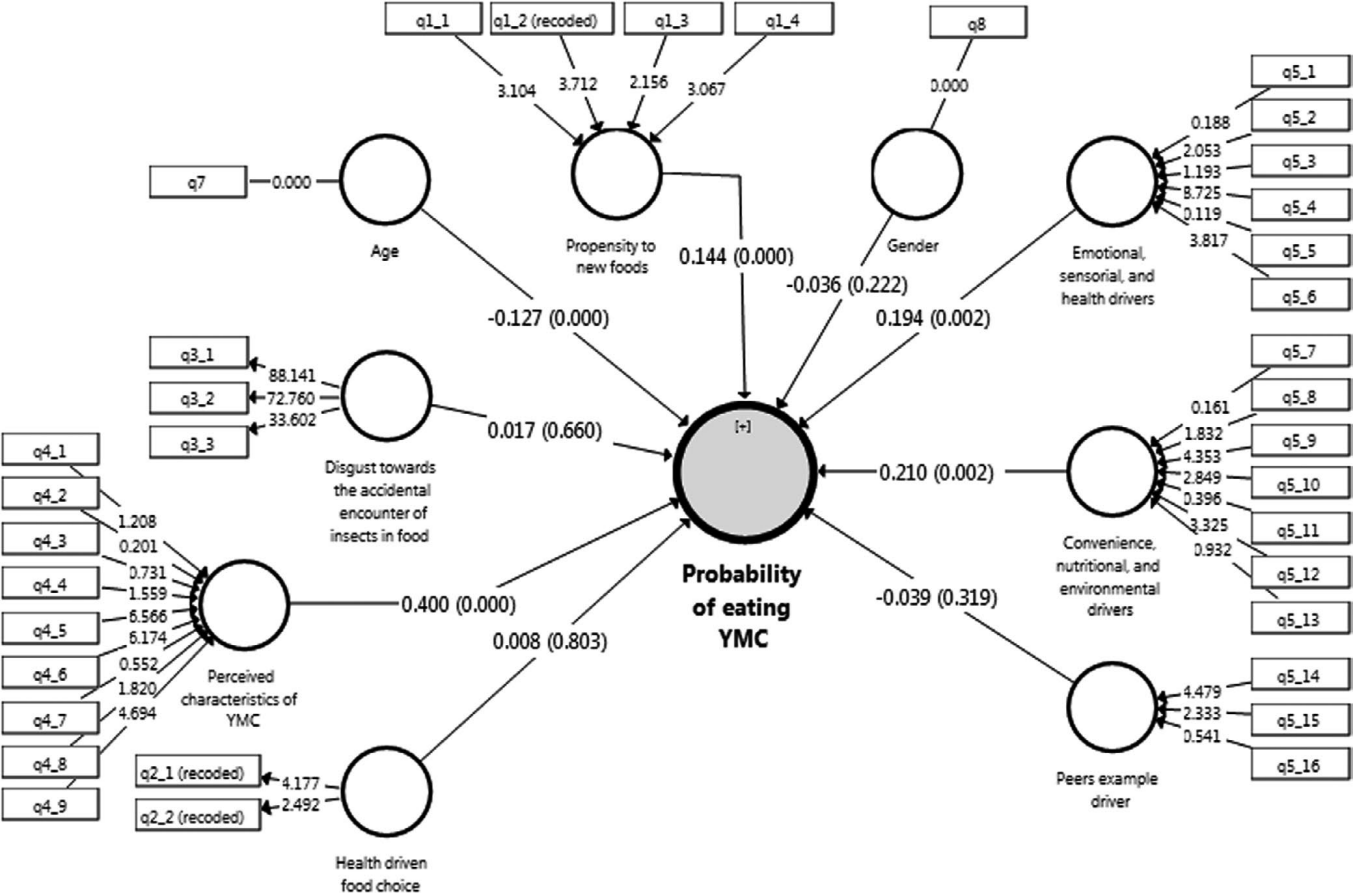
Evaluation of the structural model of the probability of eating yellow mealworm chips (YMC)

Figure 2 shows the total effects with their p‐values. The outputs of the structural model estimate are shown in Figure 2 and Table 5. We run the structural model using the bootstrap method with 500 and 5000 times of resampling and the magnitude of the structural paths was validated. The results of the model are indicated in Figures 1 and 2, showing relations between different observed and latent variables, as well as their direct impacts on the probability of eating YMC.

**TABLE 5:** Structural estimates

Path (From→to)	Path Coef.	*T* values
Age →Probability of eating YMC	−0.127***	3.681
Gender →Probability of eating YMC	−0.036^NS^	1.221
Convenience, nutritional, and environmental drivers →Probability of eating YMC	0.210**	3.113
Emotional, sensorial, and health drivers →Probability of eating YMC	0.194**	3.042
Disgust with the accidental encounter of insects in foods →Probability of eating YMC	0.017^NS^	0.440
Relationship between consumers' attitude regarding food healthiness and their food choice →Probability of eating YMC	−0.008^NS^	0.250
Peers' example driver →Probability of eating YMC	−0.039^NS^	0.997
Perceived characteristics of YMC →Probability of eating YMC	0.400***	8.143
Propensity to new foods →Probability of eating YMC	0.144***	4.704

Note

*** *p* < .001; ^**^ *p* < .01; ^*^ *p* < .1.

Abbreviations: NS: not significant; YMC: yellow mealworm chips.

The path coefficients and “*t*” values are indicated in Table 5. As can be seen, age has a direct negative (*β *= −0.127) and significant (*p* < .001; see Figure 2) effect on the probability of eating YMC. This negative coefficient path indicates that an increase in age is associated with a decrease in the probability of eating YMC.

The convenience, nutritional, and environmental drivers have a positive (*β* = 0.210) and significant (*p* < .002) effect on the probability of eating YMC. Therefore, this result indicates that an increase in this factor causes an increase in the probability of eating YMC.

Likewise, emotional, sensorial, and health drivers have a positive (*β* = 0.194) and significant (*p* < .002) effect on the probability of eating YMC. This means that an increase in the power of the emotional, sensorial, and health drivers is associated with an increase in the probability of eating YMC.

The propensity to new foods has a positive (*β* = 0.144) and significant (*p* < .001) impact on the probability of eating YMC. Therefore, when the propensity to new foods increases, the probability of eating YMC also increases.

The perceived characteristics of YMC have a positive (*β* = 0.400) and significant (*p* < .001) effect on the probability of eating YMC. This positive coefficient path shows that an increase in the perceived characteristics of YMC is associated with an increase in the probability of eating YMC. Moreover, this construct has the highest path coefficient among all other constructs of the model research. This means that the perceived characteristics of YMC are the most important construct among independent factors.

According to Table 5, some constructs do not have a significant path coefficient with the probability of eating YMC, such as disgust with the accidental encounter of insects in foods, the relationship between the attitude of consumers about food healthiness and their food choice, peers' example driver, and gender. We can conclude that these constructs do not contribute to the prediction of the probability of eating YMC.

Regarding the structural model, the PLS‐SEM method was applied to analyze the Stone–Geisser's *Q* ^2^, *R* ^2^, and path coefficients to observe the predictive accuracy and power of the model and the strength of the relationship between constructs in the determined paths. Table 6 indicates that the *Q* ^2^ value in the form of cross‐validated redundancy for the model's endogenous constructs is positive with a value of 0.591, revealing that the model has predictive accuracy (Hair et al., 2016). This value is following the same statistical measure. The *R* ^2^_adj_. obtained is 0.642, which indicates that the model has a high predictive power (Henseler & Sarstedt, 2013). Other papers that investigated the acceptance of novel food using PLS‐SEM reported similar levels for *R* ^2^_adj_. For example, Lang (2020) obtained 0.572 for the acceptance of blended food (blending mushrooms into meat foods). A study on tourists' intentions to consume ethnic Malaysian food reported *R* ^2^ values around 0.4 (Ting et al., 2019). Comparatively, the results of the presents study are better, as they show that 64.2% of the variance in the probability to eat YMC is explained by the model, while in other studies this variance is lower.

**TABLE 6:** Q^2^, *R* ^2^, and *R* ^2^_Adjusted_ of the research model

	SSO	SSE	*Q*² (= 1 − SSE/SSO)	*R* ^2^	*R* ^2^_adjusted_
Probability of eating YMC	395	161.534	0.591	0.650	0.642

Abbreviations: SSE, sum of squares of prediction errors; SSO, sum of squares of observations; YMC, yellow mealworm chips.

## 5 DISCUSSION

This study investigated consumers' attitudes toward YMC and revealed a set of variables that predicted YMC consumption. Several food‐related attitude variables, perceived characteristics of YMC, the importance of various YMC consumption drivers, and the probability of YMC consumption were investigated as components of attitude. The attitudes were investigated, since previous studies showed that attitudes were a strong predictor of the willingness to buy insect food products (Piha et al., 2018).

The findings revealed the propensity for new foods among the investigated consumers, even if its value is close to the neutral point (Table 1). In other words, food neophobia exists, but it is low. This result practically emphasizes an attitude favorable to YMC consumption. The fact that the lowest score on the “Propensity to new foods” scale was obtained by the statement “If I don't know what food contains, I don't eat it” (reverse coded in analyses) suggests that clear communication of food content can increase food acceptance, YMC in particular. In the case of YMC, this information can include details about the insect content, such as percentage per 100 g, type of processing (e.g., thermally dried and transformed into powder/grounded), and safety information such as reference to the legal document that allows its use for human consumption. At the same time, the results of the PLS analysis indicate that the propensity to new foods is one of the predictors of eating YMC. The higher the propensity to new food, the higher the probability of eating YMC. This makes the food novelty seekers a target group for YMC consumption. However, the warning of Lammers et al. (2019) is also relevant. They stress out the possibility that sensation seekers might fulfill their desire for sensation with the first attempt of eating insects and not consuming again. This possibility infers the need to offer them additional motivations to repeat consumption.

Let us look at the result according to which the respondents assigned 4.71 points (sample average) to the importance of food healthiness in their food choices in general. This shows that healthiness is, in general, important, but not very much. The score is very close to the score obtained by the relevance of healthiness as a driver of YMC consumption (Table 1). The results of the Wilcoxon Signed Ranks Test did not show a statistically significant difference between the importance of food healthiness in food choice in general and the importance of healthiness for YMC consumption (*Z* = −1.368, *p* = .171), indicating consistency in how they use healthiness in food choices. At the same time, according to the PLS analysis, this variable does not have a statistically significant power to predict YMC consumption. Similarly, Verbeke (2015) found that the importance of food healthiness in Belgians' food choices had a marginal effect on their attitude toward IBF consumption. Therefore, healthiness should not be the characteristic most promoted to consumers to stimulate their YMC consumption.

This contribution also reveals that people are disgusted when they accidentally find insects in their food; however, the disgust is not very high. At the same time, based on the PLS results, this variable does not contribute significantly to the prediction of YMC consumption. Much stronger disgust is felt when consumers see parts of yellow mealworms in the YMC. The Wilcoxon Signed Ranks Test shows a statistically significant difference between the disgust felt when they accidentally find an insect in their meal and the disgust felt when they see parts of mealworms in chips (*Z* = −11.666 (soup), *Z* = −13.072 (fresh cherries), and *Z* = −9.085 (polenta), *p* =.000). The difference can be rooted in the fact that accidental encounter refers to situations and insects that consumers are familiar with, while they are not with yellow mealworm. It was observed that familiarity was a driver for the use of food products (Verbeke, 2015). Hence, more frequent exposure to yellow mealworm as food may reduce consumer disgust and increase its acceptability. The appearance of YMC when the yellow mealworms are not visible obtained the second‐best score. The results also show that appearance is the most important consumption driver (score 4.96; Table 1). This offers a clear indication that YMC should not contain visible insect parts and that presenting them in the form (color, shape, size, texture, etc.) preferred by consumers can make them more attractive. The environmental impact obtained the best score among the characteristics of the YMC. Therefore, we can say that YMC have an environmentally friendly image in consumers' mind. This can be used as a proconsumption argument for people who care about environmental protection. Most of the remaining characteristics of YMC are perceived to be slightly below the midpoint, highlighting the need to improve people's perception of YMC. For example, adding ingredients (e.g., spices) can change the taste, odor, and texture of the YMC and make them more appealing to consumers. Overall, it is essential to improve consumers' perception of YMC because, according to the PLS analysis, they are the most important variable that predicts the probability of eating YMC.

Appearance received the highest score as a driver for YMC consumption (Table 1), and therefore great attention should be paid to the YMC aspect both on the package and on the product itself. The results of the PLS analysis indicate that the constructs “convenience, nutritional, and environmental drivers” and “emotional, sensorial, and health drivers” are both predictors of the probability of eating YMC. Consumers who attach high importance to these variables in their decision about YMC consumption have a higher probability to eat YMC. This suggests that YMC consumption probability can be boosted by increasing the importance of these variables in YMC consumption decision. For example, the importance of “nutrient intake” in consumption decision may be increased by raising awareness on specific benefits relevant to consumers. This approach can refer to the appropriate nutrient intake. Such benefits can include healthy‐looking skin, weight loss for those preoccupied with these aspects, energy input for those who feel tired, etc. The “peers' example drivers” obtained the lowest scores among all drivers, showing that, unlike other products where peers' consumption matters, in the case of YMC, seeing other people eat YMC does not stimulate consumers to eat themselves too. According to PLS results, the importance of “peers' example” in consumption decision is not predicting YMC consumption. Therefore, from a marketing perspective, it is unnecessary to invest efforts in stimulating people to consider the example of other people or to focus on advertising materials that other people eat YMC.

The consumption of YMC has an average probability (score 3.98; Table 1). This is an encouraging result, as people did not drastically reject YMC. This information is even more gratifying, as it refers to respondents who belong to a culture where insects are not consumed as food and to a market where IBF are not available in shops. Moreover, compared to other studies, these scores are relatively high. Thus, a study found that people from Finland and Sweden declared their willingness to buy IBF of 3.3 points (on a scale of 1 to 7) and people from Germany and Czech Republic showed their willingness to buy IBF of 2.7 points (on the same scale) (Piha et al., 2018). Furthermore, Verbeke (2015) found that, for a food neophobia score of 2 (on a scale from 1 to 5), 24.4% of Belgian males and 13.0% of Belgian females were ready to adopt insects as a meat substitute.

As expected, the PLS analysis shows that age indirectly influences YMC consumption. Similarly, Jaeger et al. (2021) reported that older people tend to be more neophobic than younger ones. This indicates that marketing efforts should predominantly target younger segments because they could continue their childhood feeding habits into adulthood (De Backer, 2013; Scaglioni et al., 2018). Chips are largely available, including in school vending machines, cinemas, and playgrounds in malls, but are frequently blamed for causing obesity and cardiovascular diseases (Lumanlan et al., 2020). Consequently, healthier alternatives to chips (e.g., apple, carrot chips) are often required (by parents and school educators). This makes YMC a potential healthier snack alternative to potato chips at the fingertips of young consumers.

### 5.1 Practical implications

Several practical implications can be derived from the findings suggesting effective ways to increase YMC consumption among Romanian consumers. First, the beneficial impact of YMC on the environment should be extensively communicated, as previously suggested by Hwang and Kim (2021). Specific labels and education‐information campaigns are ways to enhance potential customers' positive attitudes toward it. However, Hamerman (2016) considered that a critical mass of people cannot be reached with educational campaigns related to entomophagy, and more targeted and indirect methods of persuasion should be applied. For example, one way is to advertise YMC and IBF, in general, as gourmet, novelty, or protein‐rich foods.

Second, communication strategies should focus on the mechanisms to reframe the consumer perceptions of insects toward seeing them more as food normally consumed, blurring, thus, the connection with agricultural pests or with contaminated, expired, and decaying food. The explanation lies in the fact that people who perceive insects as dirty and as a source of contamination tend to feel a higher level of disgust for insect products (Dolezalova, 2015).

Third, food preferences and eating behaviors, mainly developed during childhood, tend to manifest in adult life (Birch, 1999), so food neophobia should be approached in early childhood. Therefore, menus in kindergartens and schools should include IBF in different familiar forms (cookies, sweet bars, chips) among familiar foods.

Finally, the inclusion of insects as ingredients in familiar and appreciated foods such as cookies and chips with preferred flavors can be another step toward their acceptance. Jones (2020) pointed out that Welsh young people were not comfortable at the thought of eating insects, but they were relieved when the products looked, smelled, and tasted familiar.

### 5.2 Research limitations

There are several limitations in this study that the authors are aware of. One refers to the representativeness of the sample, which restricts the ability to generalize the present results. However, due to the specific nature of the investigated product (YMC), which is more suitable for younger people than older ones, the selected sample can offer helpful information to stimulate YMC consumption, especially among younger consumers. The authors acknowledge that the size of the city can influence the attitude toward YMC consumption, and this was not considered in the present study. Furthermore, this paper investigated attitudes related to YMC. Therefore, it is uncertain as to what extent the present findings can be extrapolated to other insect‐based food products (e.g., crickets, waxworms). Future research could also test the actual eating behavior of YMC, as there could be a gap between the attitude toward YMC and the actual behavior, as this is often reported in the literature (Vermeir & Verbeke, 2006).

A promising future research direction is one that will focus on children. It is worth investigating the attitudes of children toward YMC (and IBF in general) and the attitude of their parents regarding the consumption of YMC by their children, since childhood feeding practices influence the individual's relationship with food (Branen & Fletcher, 1999; Nyberg et al., 2021) and chips are frequently consumed by children (Andaya et al., 2011). Therefore, a study dedicated to these groups will increase the knowledge on how to stimulate YMC consumption.

## 6 CONCLUSIONS

The present study investigated the attitude of Romanian consumers about YMC and the factors that predict YMC consumption. It was proven that Romanian consumers are interested in new foods and that the probability of eating YMC is average. This can be considered a favorable result regarding IBF consumption because it is obtained in a cultural context where insects are not perceived as food. Furthermore, the study reveals five predictors of the probability of eating YMC. Among these, the construct that matters the most in the decision of Romanian consumers to eat YMC is composed of the perceived characteristics of YMC.

Against our expectations, disgust with the accidental encounter with insects in foods does not influence the probability of eating YMC. On the contrary, a lower environmental impact compared to meat is the most appreciated characteristic of YMC, and it is also a driver that matters in the decision about YMC consumption. Therefore, a possible strategy to increase consumption likelihood is to strengthen the convenience, nutritional, and environmental drivers in the YMC consumption decision process. Furthermore, to the same end, specific environmental benefits to which target consumers are more receptive could be highlighted, such as cleaner air due to lower CO_2_ emissions or improved animal welfare by reducing slaughtered animals.

Finally, even though there are many unknowns related to insect consumption behavior at the European Union level, it is time to identify synergies on crosscutting issues of IBF, such as nutritional attributes, farm management, food safety, investment opportunities, legislation, and labeling. Last but not least, ethics should not be bypassed, as far as humans are constantly expanding the circle of moral concerns toward insects.

## CONFLICT OF INTEREST

The authors declare no conflict of interest.

## Data Availability

The data that support the findings of this study are available on request from the corresponding author.

## APPENDIX 1

**TABLE A1:** Variables used in the questionnaire and answer options

Section no	Variables and their label^a^	Items and their label^a^	Answer options
1	Propensity to new foods (opposed to food neophobia) [Propensity to new foods]	I always look for and try new foods [q1_1]	1 = Total disagreement… 4 = Neutral (No agreement, no disagreement) … 7 = Total agreement
		If I don't know what food contains, I don't eat it^b^ [q1_2]	
		At parties, meetings with friends, I try new foods [q1_3]	
		I eat almost anything [q1_4]	
2	Relationship between consumers' attitude toward food healthiness and their food choice [Health‐driven food choices]	How I choose a food does not depend on how healthy that food is^b^ [q2_1]	1 = Total disagreement… 4 = Neutral (No agreement, no disagreement) … 7 = Total agreement
		I eat whatever I like and I don't care how healthy or not food is^b^ [q2_2]	
3	Disgust toward the accidental encounter of insects in food [Disgust toward the accidental encounter of insects in food]	Worms in soup [q3_1]	1 = Totally repulsive… 4 = Neutral… 7 = Very attractive
		Worms in fresh cherries [q3_2]	
		Worms in polenta [q3_3]	
4	Perceived characteristics of YMC [Perceived characteristics of YMC]	Taste [q4_1]	1 = Horrible taste… 4 = Neutral… 7 = Very tasty
		Odor [q4_2]	1 = Very unpleasant… 4 = Neutral… 7 = Very pleasant
		Texture [q4_3]	1 = Very unpleasant… 4 = Neutral… 7 = Very pleasant
		Aspect when insect parts are visible [q4_4] (Figure A1b)	1 = Totally repulsive… 4 = Neutral… 7 = Very attractive
		Aspect when insect parts are not visible [q4_5] (Figure A1a)	1 = Totally repulsive… 4 = Neutral… 7 = Very attractive
		Characteristic of being appetizing [q4_6]	1 = Very disgusting… 4 = neutral… 7 = Very appetizing
		Nutrient intake offered [q4_7]	1 = Very low… 4 = Average level… 7 = Very high
		Consumption effect on human health [q4_8]	1 = Very harmful to human health… 4 = No effect… 7 = Very beneficial to human health
		Environmental impact compared to meat [q4_9]	1 = Much higher… 4 = The same… 7 = Much lower
5	Drivers of YMC consumption: ‐ Emotional, sensorial, and health drivers [Emotional, sensorial, and health drivers]	Taste [q5_1]	1 = Completely unimportant… 4 = Medium importance… 7 = Extremely important
		Odor [q5_2]	
		Appearance [q5_3]	
		Healthiness [q5_4]	
		Therapeutic effect [q5_5]	
		Curiosity [q5_6]	
	‐ Convenience, nutritional, and environmental drivers [Convenience, nutritional, and environmental drivers]	Affordable price [q5_7]	
		Easiness to consume [q5_8]	
		Availability in stores [q5_9]	
		Nutrient intake offered [q5_10]	
		Lower water consumption than meat [q5_11]	
		Lower environmental pollution than meat production [q5_12]	
		Reduction of number of slaughtered animals [q5_13]	
	‐ Peers' example drivers [Peers' example drivers]	My family consumes it [q5_14]	
		My friends consume it [q5_15]	
		Many other people consume it [q5_16]	
6	Probability of eating YMC [Probability of eating YMC]	Probability of eating YMC [q6] (Figure A1a)	1 = Surely not… 4 = Average probability/I don't know… 7 = Surely yes
7	Age [Age]	Age [q7]	Open answer (years)
8	Gender [Gender]	Gender [q8]	1 = M, 2 = F

^a^ The label is mentioned between “[]” and is used in Figures 1 and 2.

^b^ Reverse codes are used in analyses for these questions/statements.
